# Benchmarking Neuromorphic Hardware and Its Energy Expenditure

**DOI:** 10.3389/fnins.2022.873935

**Published:** 2022-06-02

**Authors:** Christoph Ostrau, Christian Klarhorst, Michael Thies, Ulrich Rückert

**Affiliations:** Technical Faculty, Bielefeld University, Bielefeld, Germany

**Keywords:** neuromorphic hardware, spiking neural network (SNN), benchmark, deep neural network (DNN), energy model

## Abstract

We propose and discuss a platform overarching benchmark suite for neuromorphic hardware. This suite covers benchmarks from low-level characterization to high-level application evaluation using benchmark specific metrics. With this rather broad approach we are able to compare various hardware systems including mixed-signal and fully digital neuromorphic architectures. Selected benchmarks are discussed and results for several target platforms are presented revealing characteristic differences between the various systems. Furthermore, a proposed energy model allows to combine benchmark performance metrics with energy efficiency. This model enables the prediction of the energy expenditure of a network on a target system without actually having access to it. To quantify the efficiency gap between neuromorphics and the biological paragon of the human brain, the energy model is used to estimate the energy required for a full brain simulation. This reveals that current neuromorphic systems are at least four orders of magnitude less efficient. It is argued, that even with a modern fabrication process, two to three orders of magnitude are remaining. Finally, for selected benchmarks the performance and efficiency of the neuromorphic solution is compared to standard approaches.

## 1. Introduction

With the increasing maturity of spiking neural network (SNN) simulation tools and neuromorphic hardware systems for acceleration, there is an increasing demand of potential end-users for platform comparison and performance estimation (Davies, 2019). Typical questions include the demand for speed-up of large-scale networks, potentially including plasticity rules for learning, and efficient implementations for so-called edge computing use-cases. For large-scale high-performance computing, a typical workload for comparing implementations is the full-scale cortical microcircuit model, which has been demonstrated on various platforms and forms the de-facto standard (van Albada et al., 2018; Rhodes et al., 2020; Golosio et al., 2021; Knight and Nowotny, 2021). Around the Intel Loihi chip (Davies et al., 2018) there has been a lot of work comparing SNNs to classical algorithmic approaches on standard of-the-shelf hardware systems (Davies et al., 2021). The current work is situated in between these two approaches of benchmarking individual implementations on (large scale) systems and comparing a single neuromorphic system to classical computation. It fills the gap with small to medium-scale neuromorphic benchmarks. We present our benchmark framework SNABSuite (Spiking Neural Architecture Benchmark Suite), which is publicly available. The suite currently supports simulations using NEST (Jordan et al., 2019) (CPU—single and multithreaded), GeNN (Yavuz et al., 2016) (single threaded CPU, GPU), digital (SpiNNaker; Furber et al., 2013), and analogue [Spikey; (Pfeil et al., 2013), BrainScaleS (Schmitt et al., 2017)] neuromorphic hardware. SNABSuite focuses on cross-platform benchmarking using a backend agnostic implementation of SNNs coupled to backend specific configurations (e.g., setting neuron model and parameters or network size), allowing direct cross-platform comparisons of benchmark specific performance metrics. We present results from low-level benchmarks to application related tasks like solving constraint-satisfaction problems (CSP). As an example, solving Sudoku puzzles is a representative for this class of problems and can be realized using a winner-takes-all (WTA) like implementation (Maass, 2014). This implementation is scalable, and thus it can be adapted to size constraints of neuromorphic hardware. Furthermore, different implementations of the WTA structure allow emulating the network on substrates with limited and restricted connectivity demonstrating not only how fast a system can find a solution, but also which kind of network is mappable to the system at all. Consequently, from this application a benchmark candidate for the category of computational kernel benchmarks naturally emerges: the evaluation of the various implementations of WTA networks as a building block for a broader range of applications (in addition to the CSP class of problems there is for example the spiking SLAM algorithm; Kreiser et al., 2018b). Even closer to the hardware system is the first category of benchmarks targeting lower-level features of the system and characterizing its basic properties. These properties, an example is the spike-bandwidth between neurons, are effectively limiting all networks and as such are relevant when designing a network for a specific system. They are not solely given by pure theoretical considerations but depend on several factors: runtime optimizations in the internal event routing, the chosen connectivity and combined spike rates impact these characteristics. For example, a given connectivity might fit onto the neuromorphic system, however, when operating at its limits, spike loss might still occur.

Another very common approach of using SNNs, which is applicable to all target platforms, is the conversion of pre-trained artificial networks (ANN) into SNNs (Rueckauer et al., 2017). Here, we support rate-based as well as time-to-first-spike-based encodings, and through different network layouts and sizes we are able to fully utilize the small-scale Spikey chip as well as the larger SpiNNaker system, allowing a fair comparison of key characteristics like time and energy per inference. A related sub-task is measuring the resemblance of the neuron activation curve to the ReLU function used in the ANN.

Due to a mixture of qualitative and quantitative benchmarks, the suite does not provide an oversimplifying benchmark score as known from suites in classical computation. Furthermore, a recent addition to the framework is an energy model which allows to estimate the energy expenditure of neuromorphic systems by running simulations in, e.g., GeNN or NEST on standard hardware. The estimated results closely resemble previously published values and are confirmed by newer measurements. All in all, this results in a benchmark suite which is reflecting, up to a certain extent, the current state of the art of SNN algorithms that are applicable to the aforementioned neuromorphic platforms and simulators. Hence, the suite fulfils the major requirement of being representative and relevant to our key audience of potential end-users (from neuroscience).

The remainder of this paper is structured as follows: the methods section introduces the neuromorphic systems and SNN simulators used in this work. The benchmark suite and its design are discussed as well as selected benchmark networks. Before elaborating the energy model and contributions to the energy expenditure of the human brain, selected benchmarks of the proposed suite are discussed. Results of the latter are detailed in the respective chapter. The energy model is validated using several of these benchmarks and a naive upscaling of related energy costs allows to compare the hardware systems to the human brain. Finally, the performance and efficiency is compared to classical approaches (algorithms or ANN accelerators) where applicable. The last section provides a summary and an outlook.

## 2. Methods

This section provides an overview of the employed SNN simulators and neuromorphic hardware systems before discussing the benchmark suite, selected benchmarks, and the energy model.

### 2.1. Neuromorphic Systems and Simulators

In the following, all neuromorphic systems and simulators used in this work are reviewed. A summarizing table is provided in Supplementary Table 1. When it comes to standard of-the-shelf hardware, like CPUs and GPUs, our benchmarks utilize two simulators. The **NEST** simulator (Gewaltig and Diesmann, 2007) is suited for large scale simulations of SNNs on multiple computation nodes (in HPC systems) or multithreaded simulations on a single node. The simulation code as well as the neuron models are written in C++ code and pre-compiled at installation time. Through a Python interface [PyNEST (Eppler, 2008) or PyNN (Davison, 2008)] the user can build networks of neuron populations and spike or current sources to provide input to the simulation. **GeNN** (Yavuz et al., 2016) supports both single threaded CPU simulations and GPU simulations using CUDA or OpenCL. In this work we test only an NVIDIA RTX 2070^1^, thus we stick with the CUDA backend. Similar to NEST, GeNN allows building networks within Python, but the direct interface is written in C++. Neuron and synapse models are programmed in an imperative way and are compiled at runtime. In contrast to NEST, which was created to reproduce exact spike trains with accurate simulations and hence using a fourth order Runge-Kutta-Fehlberg integrator for the LIF neuron models, GeNN uses a closed-form representation assuming a fixed input current over the integration step, which is usually set to 0.1ms. Especially with larger time steps, this can lead to numerical artifacts in the membrane voltage as well as in spike times (see Hopkins and Furber, 2015 for a discussion of the precision of various numerical solvers).

Closest specialized hardware system to CPU/GPU simulators is the **SpiNNaker** platform (Furber et al., 2013). Fabricated in a 130nm CMOS process, the SpiNNaker chip consists of 18 general purpose ARM968 cores with 16 cores being used for the simulation of spiking neurons. Each core features 64KB memory for data and 32KB for instructions, each chip has access to additional 128MB of off-die SDRAM. Several chips are connected in a toroidal way and combined to form a small scale four chip board (SpiNN3) or a 48 chip system (SpiNN5). Between cores and chips, the spike communication is using address event representation, where a single packet contains the address of the sending neuron and the spike time is modeled by the time of appearance (Furber et al., 2014). For accessing the hardware, a PyNN interface is provided which is coupled to the various components of the SpiNNaker software stack SpiNNTools (Rowley et al., 2018) and sPyNNaker (Rhodes et al., 2018). The software stack maps the individual networks at runtime to the attached system, placing at most 255 neurons on a single core. Similar to GeNN, the numerical integration is using a closed form solution for the LIF model and assumes constant currents during the full time step. When using an algorithmic timestep of 1ms, the full simulation is running in realtime, which means that 1s of model time is simulated in 1s of wall-clock time. When reducing the algorithmic timestep to 0.1ms to increase the accuracy of the simulation and to potentially reduce the amount of spikes per machine timestep, the system slows down the simulation by a factor of 10.

Finally, two mixed-signal systems from Heidelberg are used within this work. The **Spikey** system (Pfeil et al., 2013) employs above threshold analogue circuitry implemented in a 180 nm CMOS process. Spikey consists of single chip featuring two blocks of 192 neurons each supporting up to 256 independent synaptic inputs. Because of the digital communication of spikes, these neurons can be connected quite flexibly with some constraints regarding cross-chip connectivity and enforcing the separation of excitatory and inhibitory neurons. The chip emulates neurons with a fixed acceleration factor of 10, 000, which means that 1s of model time is emulated in 0.1ms. To counter the analogue mismatch between the neuron circuitry, the software interface has a built-in calibration and maps high-level parameters of LIF neurons in the PyNN interface to adapted low-level hardware parameters. Spikey emulates conductance-based LIF neurons with some restrictions on neuron parameters, and weights are encoded with 4 bit precision and a fixed range of values. As the neuron model is implemented as circuitry, there is no flexibility in changing the neuron model itself. The successor system, **BrainScaleS**, is implemented using updated HICANN chips and supports much larger networks using wafer-scale integration (Schemmel et al., 2010; Petrovici et al., 2014). The implemented neuron model is a conductance-based LIF model with an optional adaptive exponential extension. A single HICANN chip consists of 512 neuron circuits each supporting 220 synapses. Up to 64 neuron circuits (multiples of two) can be combined to form a single virtual neuron, increasing the connectivity per neuron and the robustness against noise. Wafer-scale integration is used to combine 384 accessible chips into a single addressable system, allowing to emulate networks with up to nearly 100,000 neurons. The system comes with neuron calibration to compensate for device mismatch which also provides blacklisting capabilities to exclude circuits and neurons that are not working at all or are misbehaving in some way. Other properties, like acceleration factor or fabrication technology, are similar to those of the Spikey system.

### 2.2. Benchmark Framework

**Figure 1:**
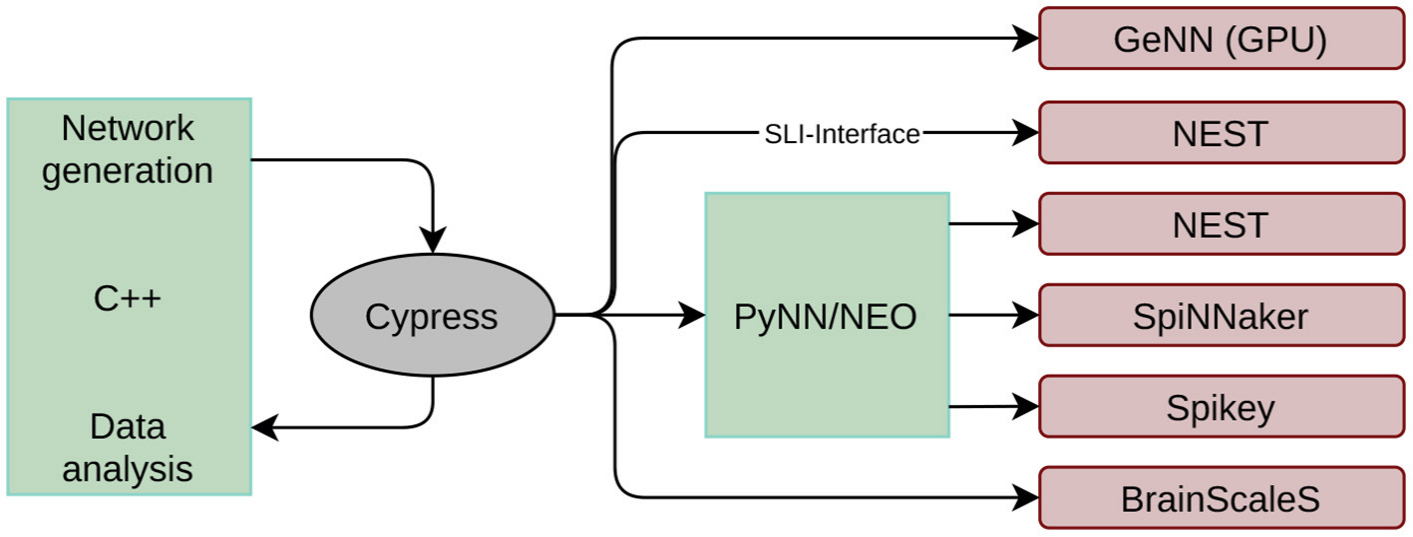
Structure of the Cypress library. Network generations and data analysis is done in C++ code. The library takes care of mapping the created network to the target platforms listed on the right side using the individual platform APIs.

The first issue encountered when developing a black-box benchmark for neuromorphic hardware is related to the various interfaces to the hardware systems. First introduced in Stöckel et al. (2017), the Cypress^2^ library is a C++ framework allowing to access all systems in a backend agnostic manner. The structure and input of an SNN is defined in an abstract interface that is quite similar to the PyNN interface. After compilation, the target backend can be chosen at runtime as long as the respective software packages (and neuromorphic systems) are installed on the working machine. Hence, network definition and data analysis can be decoupled from the actual target backend. However, it is still possible to change some low level properties at runtime, like, e.g., the number of neurons per core on SpiNNaker or the simulation time step on all digital simulators. This platform configuration is appended to the simulator string, which is provided as a command line argument, using the JSON format. At the time of writing, Cypress supports all systems reviewed in Section 2.1, and an overview of the framework is given in Figure 1. The simulation flow is the following:

- The network with its neurons and populations is set up in Cypress specific data structures.
- When run is called in the C++ source, some compatibility checks are done, e.g., regarding supported neuron models on a specific backend.
- Next, the Cypress network is mapped to the backend specific API, e.g., by creating a mirrored network in the target framework.
- Connection tables and input spikes are generated wherever a target does not natively support it.
- The backend executes the simulation and recorded data, like spikes or voltage traces, is written into the Cypress network instance.

After the simulation, spike data or voltage traces are provided in the same format and can be interpreted by the end-user.

While this abstracts away the individual backends from the main implementation of the benchmark suite, the suite itself needs to provide more flexibility regarding platform specific configuration of the benchmarks. There are two main reasons why the SNNs need to be configurable depending on the target backend: first, the different simulators/emulators support different network sizes and connectivity. Thus, to fully utilize every platform (in case of a scalable benchmark) the suite needs to include mechanisms to incorporate these properties into the configuration of the network. This is comparable to implementations of classical benchmarks as the High Performance Linpack benchmark (Dongarra et al., 2003), where problem sizes can be adapted to the target system. For neuromorphic systems, further limitations might be related to supported neuron models, restricted parameter space or bandwidth limitations. Second, the work in Stöckel et al. (2017) demonstrated that using the same neural parameters for all target platforms might give an unfair advantage to the platform for which these parameters have been tuned to. All in all, this requires us to factor out the benchmark configuration, too. Changing network sizes would usually require to recompile the whole network, which is why we included a mechanism to parse all these parameters from JSON files. Each of these benchmark specific files contain a section for every platform and the suite allows to define a default set of parameters. Configuration options depend on the individual benchmark and may include network size, neuron model and parameters or maximal spike rates. On the one hand, this allows fair comparison between different platforms. On the other hand, the actual executed workload might differ between platforms and has to be kept in mind. We see this as a compromise between real “black-box” benchmarking and individual implementations for every platform. This has been accounted for in the Spiking Neural Architecture Benchmark Suite (SNABSuite) and its architecture was already proposed in Ostrau et al. (2020b) combined with a coarse overview of the benchmark approach. Its modular structure factored out all backend specific configuration and the benchmark implementation. Furthermore, through a common API to all benchmarks, these can be interfaced by other applications e.g. for parameter sweeps optimizing the configuration, besides the mere consecutive execution of all benchmarks. To address different sizes of the systems, SNABSuite supports defining several sizes of benchmarks using the benchmark index. Thus, sizes fall into four categories: single core, single chip, small scale, and large scale system. For systems like Spikey, the first index refers to the first available category, which in this case is the single chip.

The next section will introduce selected benchmarks of the SNABSuite, called SNAB (Spiking Neural Architecture Benchmark).

### 2.3. Neuromorphic Benchmarks

**Figure 2:**
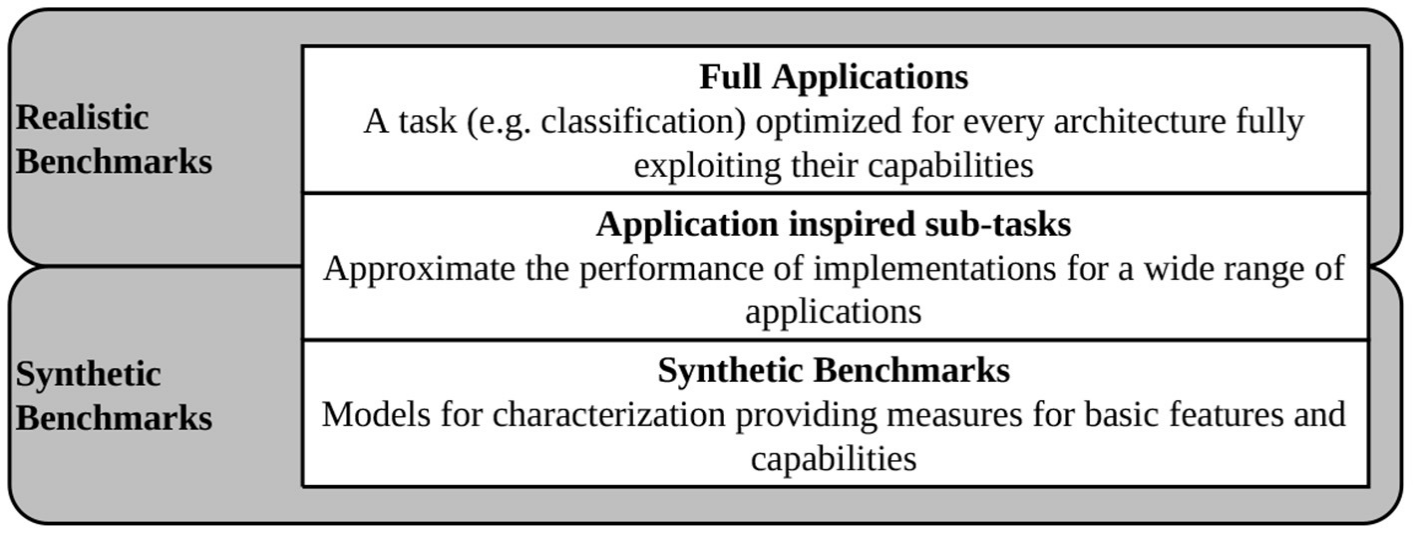
Different categories of benchmarks implemented in SNABSuite.

When choosing benchmarks for integration into SNABSuite we are limited by the main criterion: a potential benchmark has to be portable to as many of our target platforms as possible. Otherwise, the benchmark metric cannot be compared between platforms. Ideally, the potential candidate has been already successfully demonstrated. Here, it becomes clear that the implemented benchmarks can only lag behind state-of-the-art SNNs, as there is either missing support for newly introduced neuron models or learning rules (thus, these are not implemented yet or not integrated into the analogue circuitry), or there is some adaptation required for mapping the networks to the hardware. To overcome this issue at least partially, SNABSuite integrates several levels of benchmarks, categorized from low-level characterization benchmark, which measures basic hardware properties as, e.g., maximal spike rate of neurons, up to high-level application benchmarks, that measure the performance of a selected workload with reduced extrapolatory meaning to new benchmarks. These categories are described in Figure 2.

#### 2.3.1. Low-Level Characterization Benchmark

Low-level benchmarks target some basic characteristics of a target platform. The resulting performance metrics are quite universal and applicable for a wide range of applications. The first example measures the maximal output frequency of neurons. The metric of average output rate per neuron is a limiting factor in many applications, especially when using a rate encoding of data. This can have an influence on WTA networks as well, as the current winner population might spike at high rates during its winning period. For measuring the maximal output rate, neurons are put in a state where they fire by themselves by setting the resting potential above threshold. Not all simulators/emulators allow to set the reset potential above the threshold, which is why in those cases a small membrane time constant will lead to high firing rates. In addition, the output rate is limited by the configurable refractory state, providing an upper limit of the maximal measurable rate. This specific benchmark comes in several implementations, either using a single neuron, or a partially/fully recorded population of neurons. Here, the benchmark reveals whether the output rate decreases with an increased number of neurons, and whether partially recording selected neurons will have a positive influence on the measurable rate.

Other examples of this category of benchmarks include the maximal spike insertion benchmark, wherewith varying connectivity the maximal number of spikes inserted into the network can be measured, or a benchmark for spike transmission between populations.

When comparing the measured (output) rates between the platforms one has to keep in mind that these rates are evaluated in the biological time domain and do not account for the acceleration factor of, e.g., the analogue systems. Thus, a comparably small (output) rate does not immediately hint at low bandwidth between neurons on hardware.

#### 2.3.2. Application Inspired Sub-task

Here, we introduce the class of application inspired sub-tasks. These networks do not yet perform a real world application, however, they are building blocks of the latter. The aim of this kind of benchmark is to provide measures related to these applications, but having broader applicability at the same time: quite often measurements of full application benchmarks can not be extrapolated to other neural algorithms or related fields. This is where the proposed class of benchmarks steps in.

**Figure 3:**
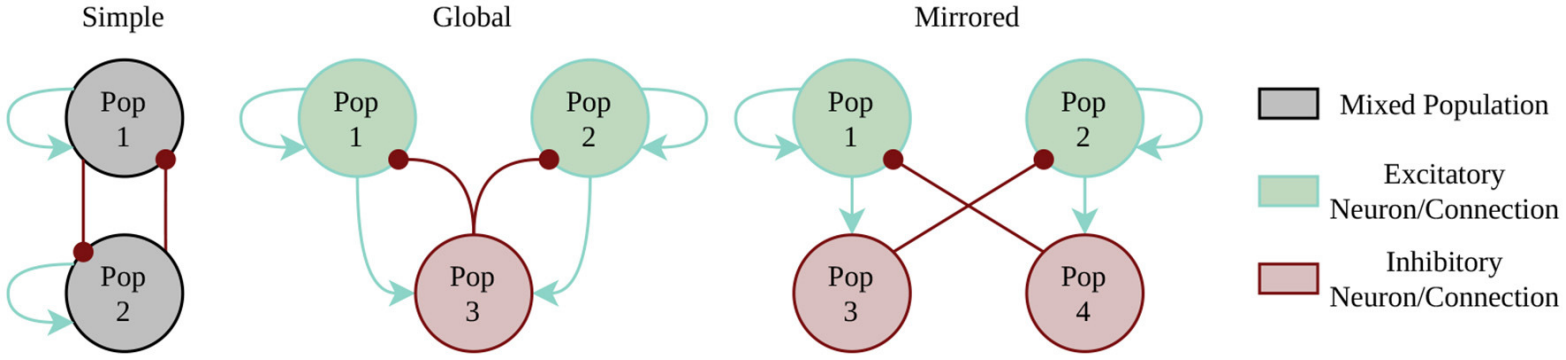
Winner-Takes-All implementation styles. Random spike source are not included in the picture, as every neuron of the excitatory populations (1 and 2) has a one-to-one connection to its individual Poisson spike source.

The first example is the class of WTA networks. WTA architectures play a major role in several tasks most notably solving constraint satisfaction problems (Maass, 2014), to implement competing behavior in self-organizing networks (e.g., Diehl and Cook, 2015) or in approaches to neuromorphic simultaneous localization and mapping (SLAM) (Kreiser et al., 2018b). Here, we test three different architectures to account for the different constraints of our target systems (compare Figure 3). The simplest instantiation of a two population WTA network uses direct cross-inhibition and self-excitation. Every population gets individual random noise via Poisson spikes source using a one-to-one connectivity scheme. However, this simplest style infringes the constraint of separating excitation and inhibition, which is mandatory for the Spikey platform. The two alternative implementations use external inhibition by either having a global inhibitory population or by using mirror neurons. Benchmark metrics include the maximal winning streak of any population, the number of state changes, and the amount of time for which no winner could be determined. These metrics allow us to qualitatively assess the performance of the WTA dynamics on a substrate by identifying too stable or too fragile winner populations. The respective winner population is determined by counting the spikes per population within a 15ms time window.

A second example is the similarity of activations curves to the rectifying linear unit (ReLU) activation function known from ANN. The motivation is clear: when converting pre-trained ANNs to SNNs a required feature is that neuron output rates increase linearly with increasing activation (Cao et al., 2015). This benchmark samples through different input spike rates measuring the output and calculating the similarity between both curves. As a main metric we chose the average deviation from the target curve for every frequency, which is then again averaged across the different frequencies. A low deviation testifies that the neural substrate is capable of reproducing the ANN activation curve. A second metric is the averaged standard deviation. Here, a high value indicates larger variances across different neurons.

#### 2.3.3. Full Application Benchmark

Full application benchmarks build the last category of benchmarks. Here, a high-level task is solving a certain problem using SNNs and benchmark metrics are usually related to accuracy. For this work we evaluated selected applications/algorithms for which the only requirement is that all target systems are able to potentially support it. This excludes networks with, e.g., continuous access to membrane potential. As an example for this category, the spiking binary associative memory benchmark (BiNAM) (Stöckel et al., 2017) calculates the retrieved amount of information in bits and compares it to the non-spiking variant. The BiNAM is trained offline and used as a synaptic connection matrix in the SNNs. Neuron parameters are tuned to reach maximal capacity, which is equal or below the capacity of the non-spiking variant. To reduce the computation time of this benchmark and in contrast to the analysis in Stöckel et al. (2017), larger networks are tested with a subset of samples only, which approximates the real relative capacity.

A second example is a spiking Sudoku solver (Ostrau et al., 2019), where the Sudokus are representative of the class of constraint-satisfaction problems. As mentioned above, this network uses WTA structures to implement the solver. Here, every possible number in the Sudoku puzzle is represented by a population of neurons. Sudoku rules, interpreted as constraints on all numbers, are implemented using inhibitory connections between the different numbers. Hence, there is inhibition between the numbers situated in a single cell, same numbers in a row, column, and sub-box of the Sudoku. Every neuron in this network has its own Poisson spike source. For analysis, spikes are binned and the respective winner in a Sudoku cell is determined. The benchmark metric is the bio-time to solution, which returns the value of the first time bin in which the solution is complete. The previous publication (Ostrau et al., 2019) analyzed the time-to-solution of 100 assorted Sudoku puzzles for every Sudoku size. Here, we reduce the analysis to a single puzzle to reduce benchmark time, but add GeNN as additional backend and compare the time and energy to solution to algorithmic approaches. Furthermore, the model is used in the validation of the proposed energy model.

A third benchmark is the conversion of deep neural networks to SNNs (Ostrau et al., 2020a). For this conversion, DNNs are trained using ReLU activation functions without biases, and for simplicity, only densely connected layers. This pre-trained network is converted to a SNN by rescaling the weights and converting the input data into rates (Diehl et al., 2015). Currently, this procedure is only evaluated for the MNIST handwritten digits dataset. To reach optimal performance, neuron parameters have to be adapted. For the analogue Spikey system, it was necessary to scale down the MNIST images by 3 × 3 average pooling to create a network that maps on the substrate. This network has as 81 × 100 × 10 layout and further employs excitatory connections only. A second network included in this analysis is the 784 × 1,200 × 1,200 × 10 network published with Diehl et al. (2015). To reach higher accuracies on the analogue system and encounter neuron to neuron variability, a hardware in the loop retraining approach is applied, similar to the one presented in Schmitt et al. (2017). Furthermore, we extended this set of benchmarks by also employing time-to-first spike encoding, where normalized input values are mapped to spikes using *f*(*x*) = (1−*x*)·*T*, where *T* is a configurable timescale. The original analysis of converted and pre-trained DNNs in Ostrau et al. (2020a) is extended here by a time-to-first spike encoding and an energy-per-inference comparison to standard accelerators for DNN inference.

**Figure 4:**
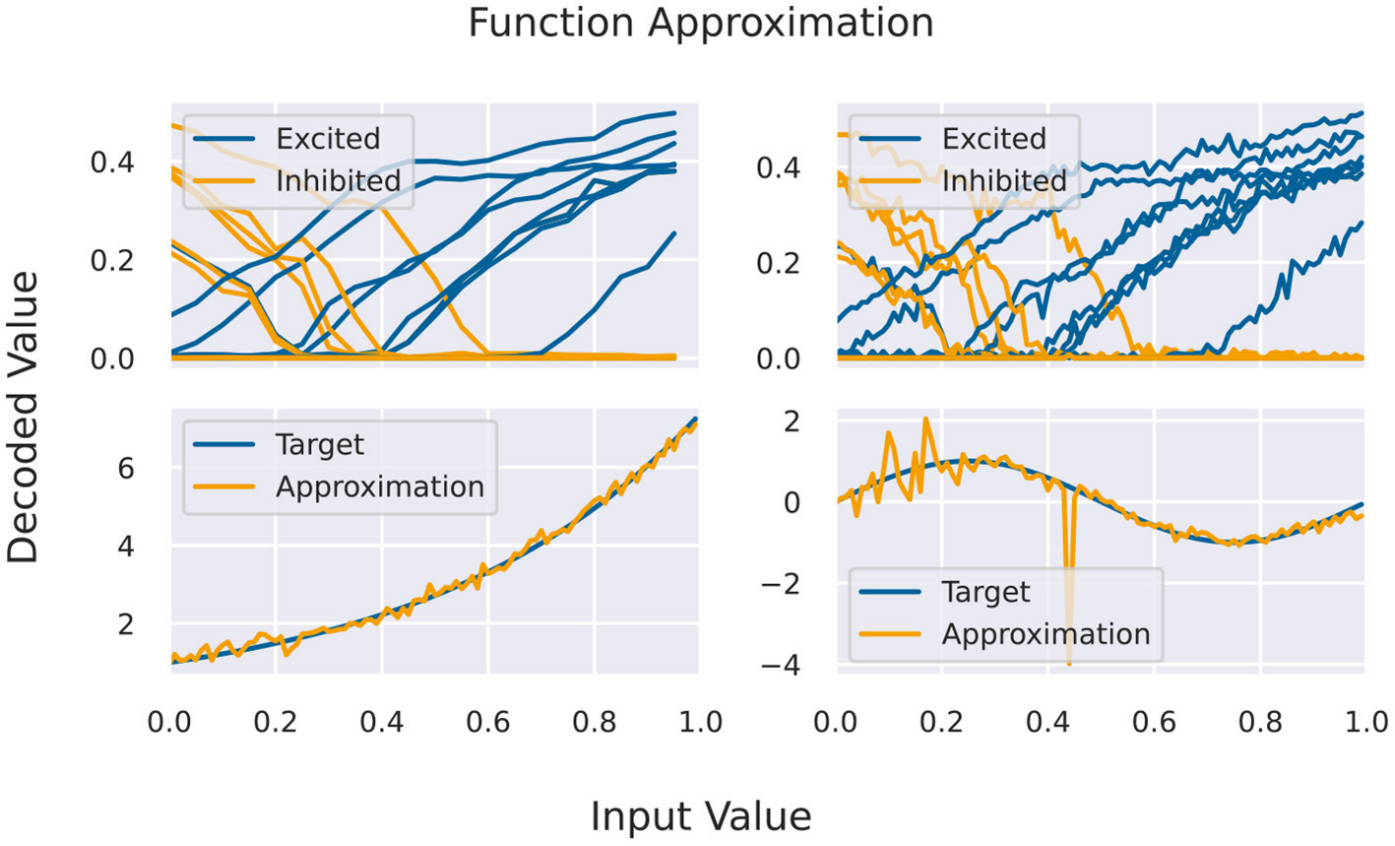
**(Top)** Activation curves for various neurons for fitting (left) and testing. Every second neuron has a positive bias, and the input is inserted via inhibitory connections (depicted in yellow). **(Bottom)** Results of the function approximation for exponential and sinus function using the testing activation curves.

A basic building block for the Neural Engineering Framework (Eliasmith and Anderson, 2004), but also an application by itself, is the approximation of functions via activation curves of neurons. For this, we feed spike rates into a population of neurons (one-to-all connection) and measure the response function of individual neurons. Given a certain variability across the population, one finds different response curves (compare Figure 4) that can be used as basis of the function space of continuous functions.


(1) $$\begin{array}{c} f(x)\approx \overset{i}{\underset{\#Neurons}{{\sum^{\text{​}}}^{\text{​}}}}a_{i}g_{i}(x) \end{array}$$

Here, *x* is a number that has to be mapped to the available rate interval, *g*_*i*_(*x*) is the decoded response rate of a neuron, and *a*_*i*_ are the neuron specific coefficients. For encoding *x* into rates, we normalize the input interval and linearly transform it to rates (given a maximal rate as a parameter). The response to that rate is decoded to values in the unit interval, again given a maximal frequency. For fixing the coefficients in Equation (1), it is required to have at least as many sampling points as neurons in the target population. For testing the approximation capabilities, more sampling points are used, including points in between the original ones for fitting, from a second simulation/emulation. This is shown in Figure 4 on the right side. Since the output activity of neurons should be more or less the same in several runs, we can use the same set of input/output rates to fit several functions. In theory, one could see the coefficients as decoding weights, and using a second matrix for encoding, we could chain these approximation populations to approximate more complex calculations, which is not currently covered in this benchmark.

An application from robotics is the spiking localization and mapping algorithm. Existing work proposes a sort of spiking state machine to track the current position and head direction of the (virtual) robot, and learning a map of the surroundings using spike-timing dependent plasticity (STDP) and a bumper sensor (Kreiser et al., 2018a,b, 2020). We adopt this network and use it as a benchmark using the accuracy of the learnt map as a benchmark metric (pixel-wise false positives for a learnt obstacle that does not exist in the simulation and false negative for a not learnt obstacle). The WTA populations used for tracking the current state of the robot could not be reliably tuned on analogue hardware using population-wise neuron parameters and would require neuron-specific tuning of 364 neurons, which is why this is currently not included. Here, only an automated approach would lead to reproducible results. The original work however targeted analogue hardware, demonstrating that the proposed algorithm is indeed suited for this kind of system.

### 2.4. Comparison to DNN Benchmarks

The original meaning of DNN benchmarks is related to datasets for benchmark DNN algorithms and network topologies, comparing the efficiency and accuracy of networks. Since our target is benchmarking hardware systems, we focus on hardware benchmarks, where the learning algorithm and network topologies are typically fixed. One of the first benchmark suites for DNN acceleration is Baidu Deep Bench^3^. This suite was introduced when the field of DNN accelerators began to grow while the DNN community was in fast progress, and representatives of an application domain are yet to exist. Thus, full applications benchmarks could not be integrated into the suite. Furthermore, every hardware system came with its own deep learning library, increasing the effort to maintain such a suite. Consequently, the authors decided to do some low-level benchmarking using typical core workloads of deep learning. These are comparable to our aforementioned low-level benchmarks. Instead of benchmarking dense or convolutional connectivity schemes or vectorized application of the activation function, our benchmark suite targets spike input and output rates, as well as the bandwidth between populations using various connectivity schemes.

Later, DAWNBench (Coleman et al., 2017, 2019) was introduced to the community. In comparison to DeepBench, the workloads cover various application categories instead of computational kernels. Besides benchmarking mere execution speed of these DNNs, its benchmark metrics account for potential differences in precision and accuracy of accelerators. As with less precise data formats the inference speed can be increased but usually at the cost of impeded accuracy. For training the network, the time required to reach a pre-defined accuracy is measured, which should account for the variances in speed and accuracy. If the criterion is met, the trained network could be used for inference, where delay between input and output is the main metric. DAWNBench built the basis for the current state-of-the-art benchmark suite MLPerf, which not only includes several application domains for DNNs, it also separates training and inference benchmarks and consists of several execution domains, from embedded to sever scale machine learning (Mattson et al., 2020; Reddi et al., 2020). In all application domains, a state-of-the-art network topology has been developed by the community, and as such, the suite can be considered to be representative of the field. Furthermore, most currently developed hardware accelerators support the feature set required to execute the selected networks. This is not the case for neuromorphic computing: There is a broad range of learning algorithms and workarounds for the back-prop algorithm, let alone the available neuron and synapse models. As such, there are only few commonly agreed learning algorithms that can be ported to all hardware platforms. Hence, our benchmark suite relies on low-level benchmarks too which, as a class of benchmarks, have been historically the first step to DNN benchmarking.

### 2.5. Energy Model for Neuromorphic Hardware

To estimate the power consumption of a network simulated on a target system we identified the following workloads as core contributions to the overall energy expenditure:

- The static power consumption of the idle neuromorphic system
- The energy required for a virtual spike source neuron to emit an event
- The power required to simulate/emulate an idle neuron
- The energy used for a real neuron to emit a spike
- The energy expenditure of transmitting a single spike
- The static power required for activating STDP and the energy per synaptic event

By subsequently activating different parts of a full network we are able to calculate the different contributions from individual processes. In more detail, we start measuring idle power consumption. Next, idle neurons are simulated. For calculating the power per simulated neuron, the idle power is subtracted. Following this line, the energy per generated action potential is measured by simulating neurons that fire by themselves after subtracting the power for idle neurons and idle hardware. In the end, the different processes are mapped to a power/energy budget. Given a network simulation, the overall energy expenditure can be calculated. For simplicity of the model, we do not account for different channels of spike communication. For example, whether source and target neuron are situated on the same chip or in close physical neighborhood plays no role in our model.

For SpiNNaker, the power measurement is done using a Ruideng UM25C USB meter. It measures the power for a supply voltage up to 20V, which allows the measurement of both the small SpiNN3 board (5V) and the larger SpiNN5 board (12V). For Spikey, the sample rate of the USB meter is insufficient. Thus, we fix the power supply (Aim TTi CPX200DP) to 5V and measure the supply current using a Fluke 289. The NVIDIA GPU allows to directly read out the current power consumption using monitoring tools. All setups allow automation of the measurement process using either the provided Bluetooth or serial interface. For the final calculation, every value is an average covering 20 measurements. Furthermore, the devices have been plugged in for several minutes to assure that devices reach their idle temperature.

To compare the energy expenditure of neuromorphic hardware to its biological counterpart, we use the data acquired by Attwell and Laughlin (2001), which calculate the amount of ATP molecules required to maintain resting potential and for active signaling. These values were calculated for the rat's neocortex and have to be adapted for the human brain. According to Lennie (2003), the human brain consists of larger neurons, which can be accounted for by using factors of 2.6 for the energy expenditure of maintaining resting potential and 3.3 for action potential generation. Finally, Howarth et al. (2012) argues that the overlap of sodium and potassium fluxes during action potential generation in the human brain is actually smaller than originally assumed, correcting the original value of 4 from Attwell and Laughlin (2001) to 1.24. Furthermore, the costs of auxiliary functions in the brain (“housekeeping”) is about 33% of the signaling costs (Howarth et al., 2012). Table 1 lists the different contributions to the overall energy expenditure of a single neuron. The resting potential expenditure includes the costs of glia cells under the assumption of a one-to-one correspondence. The contribution of “other pre-synaptic loads” includes the costs for vesicle cycling and Ca^2+^ recycling. For the overall power consumption we assume (following Attwell and Laughlin, 2001) an average spike rate of 4 Hz while the average fan out of a neuron is 2,000. For converting the amount of freed energy per ATP molecule, Rosing and Slater (1972) reports 4.6495−5.5628·10^−20^ J ATP^−1^. Hence, we used 5·10^−20^ J ATP^−1^ for our calculations. When scaling the energy expenditure up to full brain size using 8.61·10^10^±8.12·10^9^ neuron cells of the human brain (Azevedo et al., 2009) we end up with an overall energy expenditure of 21.5W.

**Table 1:** Various contributions to the overall energy expenditure of the human brain.

**Action**	**ATP molecules**	**Energy expenditure**
		**in W**
Action potential	1.57 ×10^9^	7.86 ×10^−11^
Resting potential	1.15 ×10^9^	5.77 ×10^−11^
Post-synaptic receptors	1.40 ×10^5^	7.00 ×10^−15^
Neurotransmitter recycling	1.14 ×10^4^	5.70 ×10^−16^
Other pre-synaptic loads	1.20 ×10^4^	6.00 ×10^−16^
Single neuron	4.03 ×10^9^	2.02 ×10^−10^
Single neuron + housekeeping	4.98 ×10^9^	2.49 ×10^−10^

*Single neuron refers to a spike rate of 4Hz and a fan-out of 2,000*.

## 3. Experiments and Results

This section provides results for the three categories of benchmarks. Major outcomes are discussed and evaluated. Furthermore, the proposed energy model is validated on selected benchmarks. Since individual contributions to the energy model are known, the model allows us to trivially upscale networks to brain size and compare neuromorphic to biological energy efficiency. Finally, the efficiency is also compared to algorithmic approaches for the Sudoku network and to ANN accelerators for the converted pre-trained networks.

### 3.1. Characterization Benchmarks

**Figure 5:**
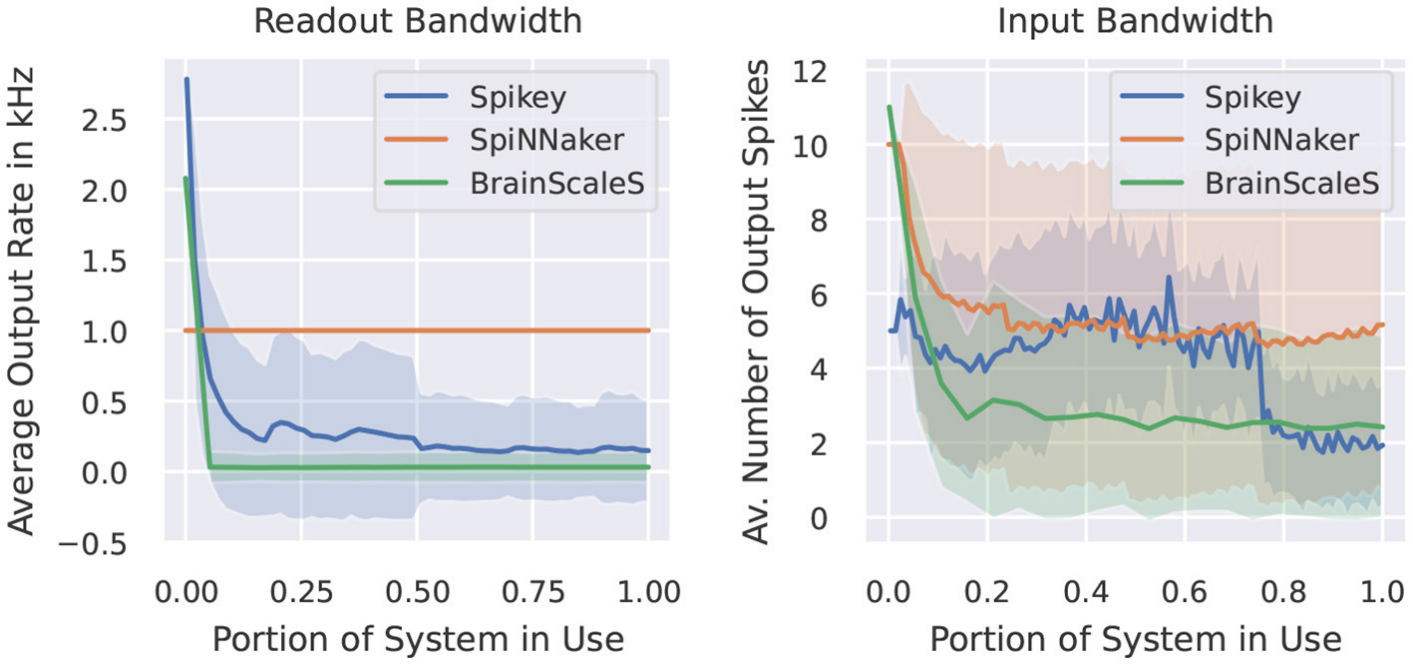
Measured output rate (left) and the indirectly measured input rate from virtual spike source neurons using a one-to-one connection scheme (right). Light coloring indicates the empirical standard deviation across neurons.

In this category of benchmarks we look at two examples. The first one measures the maximal output rate of a set of neurons. Figure 5 demonstrates the behavior of our target neuromorphic systems regarding this metric. CPU/GPU simulations are not included, as these do not suffer from any spike loss. Here, the maximal output rate is only limited by time resolution and the refractory period of the simulated model. Quite similar, the SpiNNaker system does not show any output bandwidth issues. The maximally required output buffers can be calculated before the simulation and the SpiNNaker software stack will ensure that spikes are copied to the host machine when reaching these limits. Both analogue systems suffer from bandwidth restrictions, as spikes are communicated via the on-chip network before reaching the target storage. However, the systems run in an accelerated manner, so in wall-clock terms the actual rates are increased by a factor of 10^4^.

The second benchmark indirectly measures the amount of output spikes measurable when all neurons receive a spike at the same time via one-to-one connection. Ten spikes per neuron are inserted during the simulation and we provide the average number of output spikes. On SpiNNaker, the loss usually appears at the receiving neuron. If a core (simulating 255 neurons) receives too many inputs within the same timestep, the computation of that core lags behind the global timer. In our case, the computationally more expensive conductance-based LIF neuron model requires more resources than the current-based one, thus these limits depend on the neuron/synapse model in use. Note, that if running into such problems, the SpiNNaker software stack provides several configuration options to reduce the computational load of individual cores, including a slow-down of the simulation or the reduction of simulated neurons per core (which then requires more cores for the simulation of the network). For the Spikey system, the quite prominent drop in output spikes is related to the usage of the second block of neurons of the system. When using a single block only, the average amount of spikes is more or less constant, indicating that the origin of spikes loss is not a bandwidth issue, but more related to the neuron to neuron variability. For BrainScaleS, the amount of spikes for few neurons reaches the target of 10. Here, we expect that the closeness of output spikes reaches some output bandwidth bottlenecks. The overall constant curve for larger networks indicates that there is no additional constraint when using multiple HICANN chips.

### 3.2. Application Inspired Subtasks

The WTA behavior is tested for two populations only to check the general capability of the system to demonstrate the appropriate behavior. Results for this set of benchmarks are provided in Supplementary Table 2. For analogue platforms we see in spike raster plots, that random neurons are activated quite often. Especially for Spikey one can distinguish several neurons that emit spikes more easily compared to neighboring neurons. This reduces the capability of the substrate to simulate two equally probable winner populations and acts as a bias. To account for variances across simulation due to random seeds and trial-to-trial variation in analogue systems, benchmark metrics are averaged using ten simulations. The chosen metrics for this kind of network do vary a lot across simulations, as the random input noise to neurons is different for every instance. For BrainScaleS the obtained values are comparably worse, even in the one-to-one comparison to Spikey. This is basically due to different days of the evaluation and parameter tuning leading to different results and this should not be seen as a general deficit of the hardware platform. The second part of the table (in the Supplementary Material) shows values for the exact same benchmarks, but using only as few neurons as possible [simulators: one (source) neuron; Spikey: two neurons, doubled number of source neurons; BrainScaleS: two source neurons, but four neurons per population]. Here, all platforms demonstrate the capability of embedding WTA dynamics, with analogue hardware tending to have a higher variation in winners compared to the simulators.

**Figure 6:**
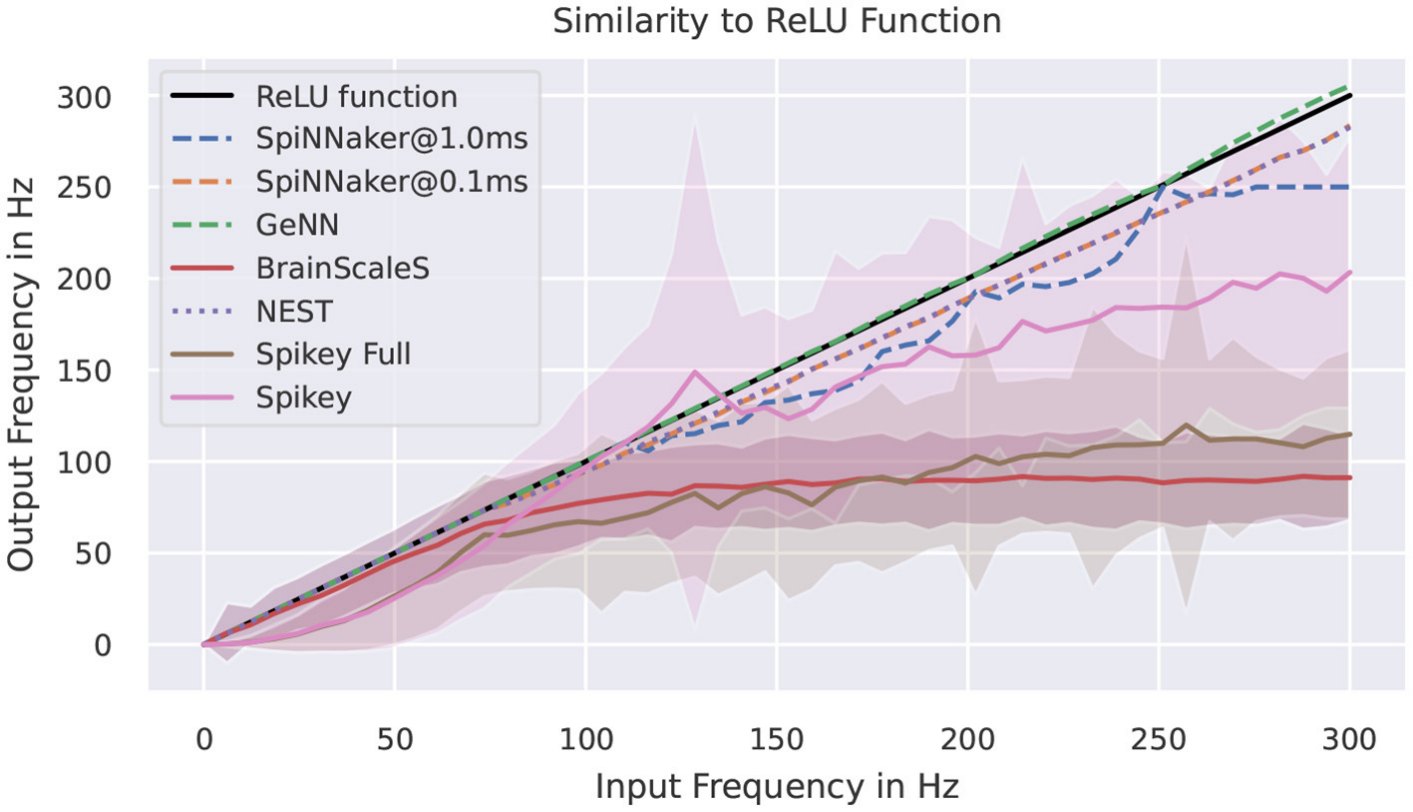
Activation curves for various simulators. “Spikey Full” refers to the usage of both neuron blocks on the Spikey system. The target curve is depicted in black. SpiNNaker is tested with two different simulation timesteps.

Figure 6 shows measured activation curves. The aim is to reuse these curves to approximate the ReLU activation function known from the field of deep learning. Thus, the aim is to measure the capability of a system to simulate converted pre-trained networks with rate-encoding. Using a single input neuron that is connected to a simulator specific number of neurons, all target systems demonstrate the ability to approximate the ReLU function in certain frequency boundaries. For simulators, this maximal frequency is, besides potential bottlenecks, limited by the refractory period and the timestep of the simulation. For SpiNNaker, no bottlenecks should be triggered in this setup, thus the deviations are results of the timestep of 1ms for running the network in realtime. The curves for the full Spikey system (employing both neuron blocks of the system) and the BrainScaleS system resemble each other. The rate limitation is a consequence of the large speedup and readout restrictions. The latter is due to shared priority encoders with limited maximal output rate of packages into the digital network effectively limiting the measurable number of spikes. If using only one neuron block of the Spikey system, the sensible range of frequencies is larger and meets expectations from the previously discussed low-level benchmarks. Putting these results into numbers, the Supplementary Table 3 of results in the Supplementary Material validates the discussed observations. Note, that by reducing the measured frequency corridor the average deviation is expected to decrease.

### 3.3. Applications

The BiNAM benchmark comes in four different implementation styles, using bursts and/or populations to represent bits in input and output. Here, we present the results from the simple variant only, although (Stöckel et al., 2017) demonstrated that the analogue platforms potentially benefit from averaging the output over several neurons. The results are provided in Supplementary Table 4, and we summarize the most important findings here. In comparison to the old publication, several things have changed in our evaluation. First, we reduced the amount of samples used in the spiking recall phase. While training the BiNAM, we still use the theoretical prediction for the optimal sample count for determining the amount of binary patterns to store. For recall, we make use of the first few randomly generated patterns only and the exact amount of samples is configurable. Since false positives and negatives are on average equally distributed across these random samples, results are still in overall accordance with the full recall. This effectively reduces the simulation times of the larger networks. Second, the new set of results include GeNN and BrainScaleS platforms. While simulators perform close to the original, non-spiking model of the BiNAM, analogue platforms show reduced accuracy. For Spikey, an increased amount of false positives demonstrate that input bottlenecks are not the problem. With an increased amount of output spikes due to false positives, a potential pitfall is that correct positives are lost while false positives are recorded. This seems to be more of an issue with the BrainScaleS system: Here, the smallest network produces no false positives, but already some false negatives, which are mostly related to the neuron to neuron variability. Higher accuracy could only be reached using a neuron specific training of parameters. Spreading the network to two or more HICANNs, the performance of the network is degraded. An increased amount of false positives implies an issue with the read-out causing the increasing number of false negatives. When using larger networks that are spread across the wafer, the amount of false negatives decreases. We can only guess that spreading the activated neurons across more HICANN reduces the average load on individual priority encoders and routers.

The Sudoku benchmark comes in three styles: the first one is how a possible end-user would program such a network. Every possible number is presented by its own population. Between populations, high-level connectors are used (e.g., all-to-all connectors) to implement the direct inhibition. The second benchmark implements exactly the same network but uses a single population which includes all neurons. Connections are realized using custom connection lists, only the random noisy input is connected via one-to-one connectors. Comparing results (see Table 2) of both implementations reveals how good the underlying software can merge neuron groups. On SpiNNaker, the individual populations are mapped to individual cores, thus every core simulates only few neurons. This highly inefficient usage leads to the larger network not being available on the SpiNNaker platform. Furthermore, the simulation tools GeNN and NEST do also benefit from such a merging of populations, as the wall clock to solution is lower. For the GeNN GPU, the larger Sudoku network would require lots of working memory at compile time, which is why it is not included. Otherwise, we see that the biological time to solution is unaffected by the implementation style (when keeping the seeds for random input generation fixed). For BrainScaleS, the simulation is evaluated ten times to measure the influence of trial-to-trial variations of the analogue substrate. For comparison, using random seed in a GeNN simulation for the generation of input noise results in a time-to-solution of 58.0 ± 53.7ms for the small and 2716.0±1869.5ms for the large Sudoku. Thus, the variance through trial-to-trial variation matches to some extent the variation due to different random input. While mean time to solution is similar between both implementation styles, the standard deviation is larger for the merged implementation. We are not aware of any specific issue that might be causing this and guess that this is related to the mapping to the hardware system: The networks is mapped to a restricted list of HICANNs, but we leave the placement of individual neurons to the BrainScaleS software stack. Between repetitions, this mapping is kept fixed. Due to the different layout of both networks we cannot assume that the same virtual neuron is placed to the same hardware neuron in both implementations. Thus, one can assume that the simple implementation style was mapped in favor of this specific Sudoku or that by revealing the internal structure of the network, the mapping reduces the amount of spike loss appearing during the emulation.

**Table 2:** Results of the Sudoku benchmark for several implementation styles and two Sudoku sizes.

**Platform**	**Bio runtime**	**Sudoku**	**Bio time to solution**	**Wall-clock time to solution**		
	**in ms**	**size**	**in ms**	**in ms**		
**Simple Sudoku**						
GeNN-CPU	5,000	2 ×2	20	0.81	±	0.06
	10,000	3 ×3	4420	3,645.26	±	219.17
GeNN-GPU	5,000	2 ×2	20	2.8	±	0.04
NEST	5,000	2 ×2	20	9.77	±	0.70
	10,000	3 ×3	2980	24,106.13	±	3,447.74
BrainScaleS	50,000	2 ×2	6264 ± 6333.54	0.63	±	0.63
SpiNNaker	5,000	2 ×2	20	200.01	±	0.00
**Simple Sudoku—single population**						
GeNN-CPU	5,000	2 ×2	20	0.44	±	0.06
	10,000	3 ×3	4420	1,212.41	±	82.48
GeNN-GPU	5,000	2 ×2	20	1.31	±	0.02
	10,000	3 ×3	4420	376.47	±	6.44
NEST	5,000	2 ×2	20	6.30	±	0.44
	10,000	3 ×3	2980	9,553.49	±	51.32
BrainScaleS	50,000	2 ×2	6780 ± 10899.87	0.68	±	1.09
SpiNNaker	5,000	2 ×2	20	200.01	±	0.00
	10,000	3 ×3	1660	16,600.33	±	0.00
**Mirrored inhibition Sudoku**						
GeNN-CPU	5,000	2 ×2	120	423.41	±	2.28
GeNN-GPU	5,000	2 ×2	120	25.26	±	2.07
NEST	5,000	2 ×2	100	52.79	±	0.25
Spikey	30,000	2 ×2	363 ± 288.60	0.04	±	0.03
SpiNNaker	5,000	2 ×2	140	4,200.17	±	0.00

*2 × 2 refers to a Sudoku featuring numbers 1–4, 3 × 3 is the standard Sudoku size*.

For the larger Sudoku network, we were not able to reliably emulated it on the BrainScaleS system. However, using neuron specific parameters and a hardware in-the-loop training should result in decreased time-to-solution and reliable solving for larger Sudokus, too. The last implementation benchmarked includes a workaround for the Spikey system avoiding direct inhibition. Here, we see that fast solving of such constraint problems is possible on analogue hardware, and find the shortest wall-clock time to solution.

Next, we evaluate rate-coded converted deep neural networks. We focus on two networks, the first being created to be mapped to the Spikey platform using a 81 × 100 × 10 layout without bias and inhibition. The input images are rescaled using 3 × 3 average pooling. The second network has been published by Diehl et al. (2015). The aim is to reach an accuracy close to the original ANN accuracy. Thus, we used the 10 first images of the training set to coarsely optimize SNN parameters using parameter sweeps. Afterwards, we increased to number of images to 100 to do a more fine-grained optimization of the most fragile parameters like the maximal frequency for encoding inputs and the scaling parameter for pre-trained weights. To fully utilize the systems, several parallel instances of the same network evaluate mutually exclusive parts of the test set of 10,000 images. Regarding the loss of accuracy during the conversion process, we find a drop in accuracy of up to 1.5% for digital platforms and the Spikey network. For the analogue systems this loss is significantly larger which can be accounted for using the HIL retraining. Nevertheless, the loss is about 5%. Note, that for this retraining the inference time per sample has been adapted to reach maximal accuracy (see last column of Table 3). When comparing simulation times, the advantages of accelerated analogue neuromorphic computing come into play. Only the GPU with massively parallel instances is on a comparable level (at a much larger power consumption).

**Table 3:** Results of pre-trained and converted DNNs.

**Platform**	**Parallel**	**Accuracy**	**Sim. time**			**Bio time/inf**.
	**instances**	**in %**	**in s**			**in ms**
**Spikey network 90.13%**						
GeNN-CPU	1	**89.11**	6.83	±	0.25	500
	100	88.87	4.29	±	0.02	500
GeNN-GPU	1	89.10	35.64	±	029	500
	100	88.87	0.70	±	0.01	500
NEST	1	88.98	86.43	±	2.09	500
	20	88.98	62.83	±	3.70	500
BrainScaleS	1	57.92 ± 5.92	0.95			900
Spikey	1	65.23 ± 0.78	**0.35**			300
SpiNNaker	1	88.41	6677.20			500
	239	88.40	235.22			500
**In-the-loop retraining**						
BrainScaleS	1	83.03	0.95			900
Spikey	1	**85.16**	**0.22**			180
**Diehl network 98.84%**						
GeNN-CPU	1	**98.85**	276.23	±	1.24	500
	36	**98.85**	325.56	±	3.12	500
GeNN-GPU	1	**98.85**	46.89	±	0.66	500
	36	**98.85**	**9.85**	**±**	**0.01**	500
NEST	1	98.82	1763.54	±	22.17	500
	53	98.82	2646.66	±	246.88	500
SpiNNaker	1	98.73	13695.06			500
	53	98.77	1724.87			500
**Diehl network (TTFS) 98.84%**						
GeNN-CPU	1	97.59	42.49	±	1.14	9.12 ± 1.02
	10	**97.60**	40.99	±	0.40	9.12 ± 1.02
GeNN-GPU	1	**97.60**	30.66	±	0.54	9.12 ± 1.02
	10	**97.60**	**4.37**	**±**	**0.02**	9.12 ± 1.02
NEST	1	97.59	540.95	±	7.54	9.94 ± 1.01
	10	97.57	581.39	±	1.80	9.94 ± 1.01
SpiNNaker	1	97.57	4817.04			**9.05** **±1.08**
	61	97.56	626.44			**9.05** **±1.08**

*Only those benchmarks in the lowest part of the table employ the sparse time-to-first-spike (TTFS) instead of rate encoding. An extended table is found in Supplementary Table 5. The best results are highlighted in bold*.

The network proposed by Diehl et al. (2015) features a smaller conversion loss. Here, the rate-coded variant suffers from up to 0.1% loss. Curiously, the GeNN simulation even improves the accuracy on one image which is most likely due to a lucky circumstance in the parameter/rate conversion process^4^. For SpiNNaker, the number of neurons per core was reduced to 180 (200 for the largest network on the SpiNN5 board). The machine timescale factor was increased to two (not for the largest network) effectively slowing down the simulation. Otherwise, the workload per core, due to the employed rate-coding, would overload and lead to lost spikes or even a break-down of the simulation. Thus, SpiNNaker is as fast as a single threaded NEST simulation, one order of magnitude slower than the GeNN CPU simulation and two orders of magnitude slower than the GPU simulation. When switching to time-to-first-spike (TTFS) encoding, the number of neurons per core is set to default while the timestep is decreased to 0.1ms slowing down the simulation by a factor of ten. This is due to the increased time precision required by this encoding. The overall loss during the conversion process is a bit larger, which might be due to the employed conductance-based synapse model [the original publication (Rueckauer and Liu, 2018) uses a simpler synapse model]. The overall response time (time between inserting the first spike of an TTFS encoded image) to the first and classifying spike in the last layer is about 9ms. The performance comparison to DNN accelerators is provided in Section 3.5.

Results for the function approximation benchmark are shown in Table 4. Simpler functions, like the linear function, can be approximated with a very small overall deviation. Furthermore, all target platform show quite similar approximation errors. For digital simulators this is realized using two distinct diversification mechanisms. First, a constant rate of spikes is fed to target neurons using random connections weights (both inhibitory and excitatory). Second, random but fixed input rates are inserted using fixed weights. On analogue hardware this is not necessary due to the naturally occurring neuron-to-neuron variability. Here, higher approximation errors are most likely related to trial-to-trial variances.

**Table 4:** Average deviations for selected functions in the function approximation benchmark.

**Platform**	***f*(*x*) = *x***	***f*(*x*) = *10+x***	***f*(*x*) = sin(2*πx*)**	***f*(*x*) = cos(2*πx*)**	***f*(*x*) = exp(2*x*)**
GeNN-CPU	0.01 ± 0.01	0.37 ± 0.42	0.30 ± 0.56	0.16 ± 0.27	0.17 ± 0.29
GeNN-GPU	0.02 ± 0.01	0.38 ± 0.41	0.16 ± 0.14	0.18 ± 0.20	0.12 ± 0.10
NEST	0.02 ± 0.02	0.68 ± 0.82	0.29 ± 0.27	0.17 ± 0.14	0.19 ± 0.22
BrainScaleS	0.14 ± 0.38	1.54 ± 3.78	0.77 ± 1.92	0.52 ± 1.44	0.67 ± 1.76
Spikey	0.05 ± 0.06	0.51 ± 0.54	0.27 ± 0.29	0.25 ± 0.26	0.24 ± 0.25
SpiNNaker	0.02 ± 0.02	0.41 ± 0.38	0.32 ± 0.63	0.29 ± 0.44	0.23 ± 0.30

**Figure 7:**
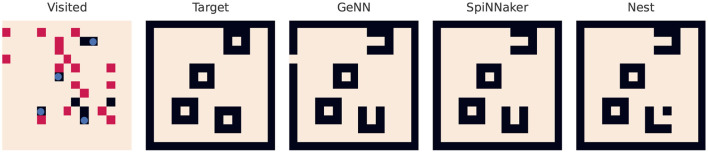
Result of the spiking SLAM benchmark. The leftmost map visualizes the map with the obstacles (blue circles). Black pixels are not visited by the virtual agent while red ones are visited once or twice. The best achievable representation is missing two pixels (see the SpiNNaker result), as two points close to the right obstacles are not visited by the virtual agent.

The last benchmark performs partial workloads of a SLAM algorithm. Figure 7 visualizes not only the coverage of the random trail of the robot in its virtual map, but also the learnt map of the simulators. This test environment features a 15 × 15 map with four obstacles. All tested platforms demonstrate the successful learning of the surroundings using the STDP (a spike pair rule with additive weight dependence) enabled connection. Small deviations from the target map occur due to not or only once visited spots in the map.

### 3.4. Energy

First, the energy expenditure of neuromorphic hardware is compared to the human brain. Table 5 summarizes costs for the various contributions to the overall energy expenditure. Values for the brain are adapted from Table 1. For neuromorphic hardware, “housekeeping” refers to the scaled system's idle power. For transmission, the values for random connectivity schemes are used. To scale values up to a full brain simulation, we assume that every neuron is connected to 2000 neurons and firing at 4Hz similar to Attwell and Laughlin (2001). The results demonstrate the superiority of the analogue system in regard to efficiency. Only the housekeeping costs are lower for the GPU due to the large number of neurons simulated. SpiNNaker performs on par with both CPUs and is less efficient compared to the GPU. Comparing the values for a single neuron or the full brain simulation, the biological paragon is four orders of magnitude more efficient than the analogue implementation. This huge difference cannot be compensated by using a modern fabrication process: According to Sun et al. (2019), the performance per watt doubles every 3–4 years. Applying the same scaling factor to the SpiNNaker system, this results in more than 8-fold improvement, which closes the gap to the GPU implementation. The step from 180 to 22/28nm FDSOI sub-threshold process would result in 50-fold improvement (Rubino et al., 2019) for analogue implementations, which most likely applies to the above-threshold circuits in Spikey, too. This results in ~2.6KW, which is still two orders of magnitude above the values found in biology. Note, that this is a naive upscaling only, as the system neither supports simulation of such many neurons nor do they provide the infrastructure to connect these.

**Table 5:** Results of the energy model in comparison to the biological counterparts.

	**Brain**	**Spikey**	**SpiNNaker**	**R2600X**	**Intel mobile**	**RTX2070**
Housekeeping	4.75E-11	1.37E-06	1.66E-04	4.49E-04	1.23E-04	**9.76E-07**
Resting potential	5.77E-11	**3.83E-08**	8.99E-05	4.77E-05	4.25E-05	3.63E-06
Action potential	1.96E-11	**4.39E-10**	1.04E-08	3.04E-08	4.46E-09	4.71E-09
Transmission	8.17E-15	**1.08E-11**	9.59E-09	5.82E-08	2.14E-08	3.40E-09
Single neuron	2.49E-10	**1.49E-06**	3.33E-04	9.62E-04	3.37E-04	3.18E-05
Full brain	2.15E+01	**1.29E+05**	2.87E+07	8.29E+07	2.90E+07	2.74E+06

*Values for the simulation of 1s of model time are reported in Joule. The single neuron and full brain estimates assume a fan-out of 2,000 synapses and a spike rate of 4Hz. R2600X: AMD Ryzen 2600X. Intel mobile: Intel Core i7-4710MQ. RTX2070: NVIDIA RTX 2070. Both CPUs are measured using a PeakTech power meter. The lowest values from simulators/emulators are highlighted in bold*.

To validate the proposed energy model, predicted values for several networks are compared to measured ones. These results are summarized in Table 6. For the Spikey system, predicted values are quite close to measured values and deviations are <10% but are not covered by the statistical error. Even better, relative deviation for the SpiNNaker system is <3%. This however is not true for the Diehl network, where low-level settings like the number of neurons have been changed explaining larger deviations. For GPU simulations the prediction is usually correct in order of magnitude, but severely deviates from actual measurements. Here, some features, like dynamic voltage and frequency scaling or temperature dependent clock rates, are not covered by the proposed energy model. Nevertheless, for Spikey and SpiNNaker the proposed model predicts the energy expenditure of a network simulation even though the network has been executed with, e.g., the GeNN simulator. More interestingly, there is an overall agreement of predictions based on analogue emulation and digital simulation. Thus, one can use the Spikey system to estimate the energy expenditure of a SpiNNaker simulation and vice versa (if the network maps to both systems).

**Table 6:** Validation of the energy model.

**Platform**	**Acc**.	**E/Inf**.	**Prediction of E/Inference in mJ**			
	**in %**	**in mJ**	**GeNN**	**Spikey**	**SpiNN3**	**SpiNN5**
**Spikey network with parallelism 1**						
GPU	86.04	160.8	46.8 ± 1.3	0.19 ± 0.00	939.9 ± 2.5	8071.8 ± 16.6
Spikey	68.89	0.2	–	0.19 ± 0.00	939.9 ± 2.5	8071.7 ± 16.5
SpiNN3	87.07	950.8	–	0.19 ± 0.00	939.9 ± 2.5	8071.7 ± 16.5
SpiNN5	87.07	8148.1	–	0.19 ± 0.01	939.9 ± 2.5	8071.8 ± 16.6
**Spikey network with parallelism 239**						
CPU	86.03	–	–	0.00 ± 0.00	89.6 ± 1.3	38.6 ± 6.4
SpiNN5	87.04	38.2	–	0.00 ± 0.00	89.4 ± 1.3	38.5 ± 6.3
**Diehl network with parallelism 4**						
GPU	98.83	217.0	265.4 ± 16.3	–	1871.1 ± 43.1	7232.5 ± 205.5
SpiNN3	98.73	993.5	–	–	1806.0 ± 41.1	7181.3 ± 196.5
SpiNN5	98.74	6597.8	–	–	1806.0 ± 41.1	7181.3 ± 196.5
**Diehl network with parallelism 53**						
CPU	98.86	–	–	–	1046.1 ± 39.6	1013.5 ± 185.4
SpiNN5	98.77	488.5	–	–	979.8 ± 37.6	961.3 ± 176.1
	—	**in J**	**in J**	**in mJ**	**in J**	**in J**
**Mirror Inhibition**		
GeNN-GPU		56.0	378.5 ± 57.0	2.7 ± 0.0	14.2 ± 59.8	160.3 ± 71.3
GeNN-GPU†		62.6	261.2 ± 82.8	2.8 ± 0.0	88.0 ± 22.9	187.8 ± 114.7
Spikey		0.0029	–	2.7 ± 0.0	155.7± 6.5	1174.1 ± 33.4
**Single population**						
GeNN-GPU		15.3	7.5 ± 0.2	2.9 ± 0.1	134.7 ± 0.4	1153.7 ± 2.4
SpiNN3		134.3	–	2.9 ± 0.1	134.7 ± 0.4	1153.7 ± 2.4
SpiNN5		1171.6	–	2.9 ± 0.1	134.7 ± 0.4	1153.7 ± 2.4
**Spiking SLAM**						
GeNN-GPU		303.1	108.8 ± 2.8	–	45.7 ± 0.12	390.6 ± 0.8
SpiNN3		46.0	–	–	45.7 ± 0.12	390.6 ± 0.8
SpiNN5		398.0	–	–	45.7 ± 0.12	390.6 ± 0.8

*For selected networks the measured energy (3rd column) is compared to the prediction of the energy model (right part of the table). For the Spikey network, input rates have been adapted to values used for the Spikey system to improve comparability. Thus, the reported accuracy might be reduced compared to Table 3. Missing values are either due to missing runtime for the GPU simulation, or due to the network not being compatible with the Spikey system. More data points are found in Supplementary Table 6*.

*^†^Simulation using three neurons per population similar to the Spikey parameter set*.

### 3.5. Comparison to Classical Solutions

Finally, we address the comparison to classical algorithmic approaches for solving a Sudoku or ANN inference. In Table 7, the time and energy to solution of the former application are compared between a Raspberry Pi 4 with 2GB of RAM and neuromorphic systems. On the Pi 4 the Coin-Or Cbc^5^ was employed to efficiently solve the Sudoku. For the small Sudoku puzzle, Spikey is the most efficient platform in regard to both time and energy to solution. The GPU is faster, but also more energy consuming compared to the algorithmic implementation. For the larger Sudoku, the latter outperforms SpiNNaker and the GPU implementation. This is most likely due to the SpiNNaker system and the GPU not being fully utilized. With a more up-to-date manufacturing process (see Section 4 above), the SpiNNaker implementation would be on the same level of efficiency as the RPI 4.

**Table 7:** Comparing Sudoku solving on a Raspberry Pi 4 using an algorithmic approach with SNN solvers.

**System**	**Time to solution**	**Energy to solution**
	**in ms**	**in J**
**2** **×2 Sudoku**		
RPI 4 2GB	5.00	0.016
SpiNN3	200.00	0.537
Spikey	**0.04**	10^−4^ × **2.105**
GeNN-GPU	1.42	0.061
**3** **×3 Sudoku**		
RPI 4 2GB	**261.0**	**0.91**
SpinNN3	560.0	16.77
GeNN-GPU	370.6	26.72

*Metrics are time and energy to solution. The fastest and most efficient values are highlighted in bold*.

For deep network inference (see Table 8), the Spikey system is again the fastest and most efficient system. However, the ANN accelerators perform similar at a higher accuracy. Again, the rather old technology in Spikey is accountable for at least an order of magnitude in efficiency. The Intel Neural Compute Stick 2 (NCS)^6^ benefits from a larger batchsize, which is defined at compile time. For the Edge TPU^7^ a batchsize could not be configured. Both accelerators were able to simulate the full network without doing computation on the host machine. The GPU simulation requires one order of magnitude more energy, while SpiNNaker (even with full utilization) requires two orders of magnitude more energy. For the larger Diehl network, SpiNNaker, and the GPU simulation are on the same level of efficiency, while the latter being significantly faster. Switching to TTFS encoding and only counting the energy expenditure until the first, classifying spike appears closes the gap between both platforms and ANN accelerators. Here, a modern manufacturing process would result in SpiNNaker being the most efficient system. Note, that the slightly reduced accuracy in SNN simulations with TTFS encoding might be encountered by using SNN specific training methods (e.g., Neftci et al., 2019).

**Table 8:** The table reports time and energy per inference in SNNs compared to ANN accelerators.

**System**	**Batch-**	**Parallel**	**Accuracy in %**		**Time per Inf. in ms**		**E per Inf. in mJ**	
	**size**	**netw**.	**value**	**to Spikey**	**value**	**to Spikey**	**value**	**to Spikey**
**Spikey network (90.13% ANN accuracy)**								
Coral edge TPU	1		**90.20**	5.04	0.05	0.03	0.3	0.1
Intel NCS 2	1		90.10	4.94	1.92	1.90	10.6	10.4
	200		90.10	4.94	0.12	0.10	0.6	0.4
GeNN-GPU		100	88.87	3.71	0.07	0.05	3.7	3.5
SpiNNaker		239	88.40	3.24	23.50	23.48	38.2	38.0
Spikey		1	85.16	0.00	**0.02**	0.00	**0.2**	0.0
				**to SpiNN**		**to SpiNN**		**to SpiNN**
**Diehl network (98.84% ANN accuracy)**								
Coral edge TPU	1		**98.85**	1.29	1.43	−4.83	7.7	3.9
Intel NCS 2	1		98.84	1.28	2.53	−3.73	13.8	10.0
	200		98.84	1.28	0.71	−5.55	**3.8**	0.0
GeNN-GPU		36	**98.85**	1.29	1.00	−5.26	181.6	177.8
SpiNNaker		53	98.77	1.21	172.49	166.23	188.5	184.7
GENN-GPU (TTFS)		10	97.60	0.04	**0.44**	−5.82	4.7	0.9
SpiNNaker (TTFS)		61	97.56	0.00	6.26	0.00	**3.8**	0.0

*The batchsize is configured for ANN accelerators. The number of parallel instances of the same network is used for SNN simulations. Values are compared to the most efficient neuromorphic solution, too. Highlighted are the most accurate and the fastest/most efficient results*.

## 4. Discussion

To tackle the problem of missing cross-platform performance assessment in neuromorphic computing, we presented SNABSuite, an open-source benchmark suite. SNABSuite features a set of workloads implemented in a platform-agnostic way, but also providing mechanisms for benchmark and platform configuration. This allows to account for varying neuron models, parameter inaccuracies, and platform sizes. The suite has been deployed to a range of neuromorphic systems (from mixed-signal to fully digital) and SNN simulators, demonstrating the capabilities of the framework. Selected benchmarks have been presented and evaluated revealing hardware specific constraints for neural modeling and potential workarounds for issues encountered when using these systems. Benchmarks belong to three categories with varying closeness to full applications and extrapolation capabilities: low-level benchmarks revealed constraints that influence the available SNNs deployable to a given system. These constraints hold for every application, thus their relevance is quite broad. Application kernels, like the presented WTA architectures, represent a full class of networks, but still not solve a real task like object detection or CSP solving. These belong to the class of full application benchmarks, providing natural benchmark metrics but having only limited meaning for other applications implemented on neuromorphic hardware. We presented results for DNN inference, function approximation, spiking Sudoku solving, and SLAM. More results can be found in the Supplementary Material.

For future development of our benchmark framework, two directions are possible: due to the modular structure and the hardware abstraction layer, adding new platforms to the comparison is eased up to a certain extent. Here, possible candidates include Intel Loihi (Davies et al., 2018), BrainDrop (Neckar et al., 2019), or DYNAPs (Moradi et al., 2018). Furthermore, successors have been announced for systems discussed here (Billaudelle et al., 2019; Mayr et al., 2019). The second direction covers the implementation of new benchmarks. Most interesting is the embedding of the various direct training methods published within the recent years. These methods allow, similar to the hardware-in-the-loop approach discussed above, to encounter the neuron variability found in analogue circuitry.

One major argument for neuromorphic computing is the improvement in efficiency compared to algorithmic or standard DNN implementations. To validate this argument, we proposed a simple energy model relating costs of high-level SNN operations (e.g., action potential generation) to low-level energy costs. This model successfully predicts the energy budget of networks emulated on Spikey or simulated on SpiNNaker as long as low-level configurations would not deviate from the default. Here, especially changing the number of neurons per core on the SpiNNaker system leads to larger deviations, as the idle cost per neuron is increased. The energy model does not cover all features of a modern digital processor, thus energy predictions for GPU simulations were found to be insufficient. Nevertheless, the model allowed us to scale up the energy budget to a full brain simulation. Comparing these to the costs of the human brain we found mixed-signal hardware, being the most of efficient system in consideration, to lag behind by four orders of magnitude (even in this very optimistic and simplified upscaling). Furthermore, switching to a modernized fabrication technology, this gap cannot be closed in the short run. To conclude the energy related discussion, we presented a comparison to ANN accelerators and to a Raspberry Pi 4 for selected benchmarks. We demonstrated, that for small scaled Sudokus the SpiNNaker system was not fully utilized and thus the RPI4 is performing better. Only the mixed-signal system had superior time and energy to solution metrics. Similarly, this system is most efficient at DNN inference at the cost of accuracy. For SpiNNaker, switching to TTFS encoding resulted in an efficiency competitive to ANN accelerators, despite the rather old technology in which SpiNNaker cores are fabricated in.

## Data Availability Statement

Publicly available datasets were analyzed in this study. This data can be found at: http://yann.lecun.com/exdb/mnist/; https://github.com/dannyneil/spiking_relu_conversion.

## Author Contributions

CO and CK conducted the experiments. MT and UR supervised this work and contributed with various corrections and comments. All authors contributed to writing the article and approved the submitted version.

## Funding

This research leading to these results has received funding from the European Union Seventh Framework Programme (FP7) under grant agreement no 604102 and the EU's Horizon 2020 research and innovation programme under grant agreements Nos. 720270 and 785907 (Human Brain Project, HBP). It has been further supported by the Cluster of Excellence Cognitive Interaction Technology CITEC (EXC 277) at Bielefeld University, which was funded by the German Research Foundation (DFG). We acknowledge support for the publication costs by the Open Access Publication Fund of Bielefeld University and the Deutsche Forschungsgemeinschaft (DFG).

## Conflict of Interest

The authors declare that the research was conducted in the absence of any commercial or financial relationships that could be construed as a potential conflict of interest.

## Publisher's Note

All claims expressed in this article are solely those of the authors and do not necessarily represent those of their affiliated organizations, or those of the publisher, the editors and the reviewers. Any product that may be evaluated in this article, or claim that may be made by its manufacturer, is not guaranteed or endorsed by the publisher.

## Footnotes

^1^Details of the NVIDIA RTX 2070 can be found at https://www.nvidia.com/en-me/geforce/graphics-cards/rtx-2070/.

^2^ https://github.com/hbp-unibi/Cypress

^3^https://github.com/baidu-research/DeepBench (accessed November 3, 2021).

^4^This is reproduced in the quantization process for the neural compute stick in Table 8.

^5^Found at https://github.com/coin-or/Cbc.

^6^Results created with Open Vino 2021.3.

^7^The TPU was interfaced with the Pycoral Frogfish Release from February 2021.
