# Supplementary material for: Benchmarking Neuromorphic Hardware and Its Energy Expenditure
Source: Front Neurosci. 2022 Jun 2;16:873935. doi: 10.3389/fnins.2022.873935 (PMC9201569; doi:10.3389/fnins.2022.873935)
Supplement: Supplementary file 1 [file Data_Sheet_1.PDF]

Supplementary Material

Table S1. Neuromporphic target systems and simulators and the specifications. Values for NEST (GeNN) are based on the AMD Ryzen 2600X (Nvidia RTX 2070).

System	Feature in nm	Die size	Neurons	Synapses	Bit per Synapse	ESE in J	Models
Nest	12	$213\,mm^2$			64		Flexible
GeNN	12	$445\,mm^2$			32 (64)		Flexible
SpiNNaker	130	$1.02\,cm^2$	≈4080	4080×10^4	8	10^{-8}	Flexible
Spikey	180	$25\,mm^2$	384	256×384	4	10^{-10}	cond.-based IaF
HICANN	180	$0.5\,cm^2$	8-512	224×512	4	10^{-10}	cond.-based AdEx IaF

Table S2. Results for the Winner-Takes-All benchmark for one implementation style using mirrored inhibition. Metrics are the maximal winning streak, the number of state changes and the time spent without a clear winning population. The lower part of the table provides results with using as few neurons as possible: Simulators use one neuron per population. Spikey requires 2 neurons per population (excitatory and inhibitory), but 4 input neurons connected to each of the two excitatory populations. On BrainScales, this required at least 4 neurons per population, with two neurons in the input.

Platform	Max Win Streak in ms	#State Changes	Time w/o Win in ms
GeNN-CPU	8739.00 ± 2463.33	2.20 ± 4.08	24.00 ± 37.55
GeNN-GPU	9153.00 ± 1480.03	1.80 ± 2.39	19.50 ± 24.55
NEST	7167.00 ± 2528.33	5.60 ± 8.91	75.00 ± 114.89
BrainScaleS	58.50 ± 11.07	261.60 ± 16.45	7446.00 ± 205.45
Spikey	3279.00 ± 1210.89	16.20 ± 6.36	97.50 ± 50.12
SpiNNaker	1855.50 ± 1094.56	67.30 ± 54.39	576.00 ± 502.25
Mirror Inhibition – Small			
GeNN-CPU	6573.00 ± 2106.12	3.50 ± 2.01	486.00 ± 442.90
GeNN-GPU	6573.00 ± 2106.12	3.50 ± 2.01	486.00 ± 442.90
NEST	2275.50 ± 512.59	23.70 ± 4.03	817.50 ± 236.88
BrainScaleS	726.00 ± 223.74	115.30 ± 9.07	907.50 ± 76.57
Spikey	2641.50 ± 1123.20	29.60 ± 9.81	442.50 ± 228.39
SpiNNaker	2955.00 ± 1310.63	16.60 ± 4.84	250.50 ± 119.80

Table S3. Results for the ReLU similarity benchmark. The benchmark metric is the deviation of the measured activation curve to the target curved averaged over all sample points. Larger values on SpiNNaker are due to the enlarged timestep (and thus accuracy of the simulation) compared to GeNN and NEST. For analogue platforms the maximally tested rate was reduced from 300 Hz to 150 Hz (Spikey) and 60 Hz (BrainScaleS).

Platform	Neurons	Av. Dev.
GeNN-CPU	100	1.28 ± 00.00
GeNN-GPU	100	1.28 ± 00.00
NEST	100	7.97 ± 00.00
BrainScaleS	56	6.56 ± 17.52
	3155	27.08 ± 31.49
Spikey	192	17.67 ± 32.24
SpiNNaker	255	13.51 ± 00.00
	3825	13.53 ± 00.00
	15810	13.53 ± 00.00
	60000	13.53 ± 00.00

Table S4. Results for the BINAM benchmark. To reduce computation time, larger networks recall only a subset of stored patterns, which is provided in parentheses if applicable. Benchmark metrics are the normed information capacity, false positives and negatives relative to values found in the non-spiking variant.

Platform	Inp Dim	Out Dim	#Rec. S.	Rel. Info	Norm. FP	Norm. FN
GeNN-CPU	100	100	527	0.995	-0.01	0.01
	500	500	500 (11,570)	0.986	-0.01	0.01
	800	800	1000 (29,002)	0.980	-0.01	0.02
GeNN-GPU	100	100	527	0.995	-0.01	0.01
	500	500	500 (11,570)	0.986	-0.01	0.01
	800	800	1000 (29,002)	0.980	-0.01	0.02
NEST	100	100	527	0.991	-0.01	0.01
	500	500	500 (11,570)	0.982	-0.01	0.02
	800	800	1000 (29,002	0.973	-0.02	0.02
BrainScaleS	50	40	117	0.472	0.02	0.38
	50	56	158	0.205	0.30	0.51
	50	344	813	0.240	0.17	0.45
	50	3408	7470	0.391	0.13	0.27
Spikey	128	192	1215	0.499	0.18	0.09
	256	384	4619	0.483	0.16	0.13
SpiNNaker	250	250	250 (3024)	0.991	-0.01	0.01
	4000	4000	500 (700,357)	0.982	-0.01	0.02
	4000	15810	500 (2,747,068)	0.983	-0.01	0.02
	50	180000	500 (389,988)	0.996	-0.00	0.00

Table S5. Complete table of results of pre-trained and converted DNNs (see Table 3 in the main text.

Platform	Parallel Instances	Accuracy in %	Sim. Time in s	Bio Time/Inf. in ms
		Spikey Network 90.13%		
GeNN-CPU	1	**89.11**	6.83±0.25	500
	100	88.87	4.29±0.02	500
GeNN-GPU	1	89.10	35.64±029	500
	100	88.87	0.70±0.01	500
NEST	1	88.98	86.43±2.09	500
	4	88.98	64.76±2.14	500
	20	88.98	62.83±3.70	500
	200	88.98	151.20±5.15	500
SpiNNaker	1	88.41	6677.20	500
	4	88.41	1771.75	500
	21	88.41	572.53	500
	239	88.40	235.22	500
BrainScaleS	1	57.92 ± 5.92	0.95	900
BrainScaleS ITL	1	83.03	0.95	900
Spikey	1	65.23 ± 0.78	0.35	300
Spikey ITL	1	85.16	**0.22**	180
		Diehl Network 98.84%		
GeNN-CPU	1	**98.85**	276.23±1.24	500
	36	**98.85**	325.56±3.12	500
GeNN-GPU	1	**98.85**	46.89±0.66	500
	36	**98.85**	**9.85±0.01**	500
NEST	1	98.82	1763.54±22.17	500
	4	98.82	1717.57±72.55	500
	53	98.82	2646.66±246.88	500
SpiNNaker	1	98.73	13695.06	500
	4	98.74	4537.52	500
	53	98.77	1724.87	500
		Diehl Network (TTFS) 98.84%		
GeNN-CPU	1	97.59	42.49±1.14	9.12 ± 1.02
	10	**97.60**	40.99±0.40	9.12 ± 1.02
	100	97.58	55.51±1.09	9.11 ± 1.02
GeNN-GPU	1	**97.60**	30.66±0.54	9.12 ± 1.02
	10	**97.60**	**4.37±0.02**	9.12 ± 1.02
NEST	1	97.59	540.95±7.54	9.94 ± 1.01
	10	97.57	581.39±1.80	9.94 ± 1.01
SpiNNaker	1	97.57	4817.04	**9.05 ± 1.08**
	5	97.55	1051.37	**9.05 ± 1.08**
	61	97.56	626.44	**9.05 ± 1.08**

Table S6. Complete table of results for the validation of the energy model using pre-trained deep neural networks (see Table 6 in the main text).

Platform	Acc. in %	E/Inf. in mJ	Prediction of E/Inference in mJ GeNN	Spikey	SpiNN3	SpiNN5
**Spikey Network with parallelism 1**						
GPU	86.04	160.8	46.8±1.3	0.19±0.00	939.9±2.5	8071.8±16.6
Spikey	68.89	0.2	-	0.19±0.00	939.9±2.5	8071.7±16.5
SpiNN3	87.07	950.8	-	0.19±0.00	939.9±2.5	8071.7±16.5
SpiNN5	87.07	8148.1	-	0.19±0.01	939.9±2.5	8071.8±16.6
**Spikey Network with parallelism 21**						
GPU	85.98	6.6	3.3 ±0.1	0.01±0.00	49.9±0.2	391.9± 1.3
SpiNN3	87.07	49.2	-	0.01±0.00	49.9±0.2	391.9±1.3
SpiNN5	87.07	393.5	-	0.01±0.00	49.9±0.2	391.9±1.3
**Spikey Network with parallelism 100**						
GPU	88.87	3.7	16.2±0.1	0.00±0.00	14.4±0.1	85.3±0.7
SpiNN5	87.06	85.4	-	0.00±0.00	14.3±0.1	85.3±0.7
**Spikey Network with parallelism 239**						
CPU	86.03	-	-	0.00±0.00	89.6±1.3	38.6±6.4
SpiNN5	87.04	38.2	-	0.00±0.00	89.4±1.3	38.5±6.3
**Diehl Network with parallelism 4**						
GPU	98.83	217.0	265.4±16.3	-	1871.1±43.1	7232.5±205.5
SpiNN3	98.73	993.5	-	-	1806.0±41.1	7181.3±196.5
SpiNN5	98.74	6597.8	-	-	1806.0±41.1	7181.3±196.5
**Diehl Network with parallelism 36**						
GPU	98.85	181.6	251.8±16.0	-	1059.4±39.6	1127.7±185.6
SpiNN5	98.75	590.0	-	-	993.1±37.6	1075.5±176.4
**Diehl Network with parallelism 53**						
CPU	98.86	-	-	-	1046.1±39.6	1013.5±185.4
SpiNN5	98.77	488.5	-	-	979.8±37.6	961.3±176.1
